# ﻿Four new species of *Symphylella* (Symphyla, Scolopendrellidae) from Chongqing, southwest China with DNA barcoding analysis

**DOI:** 10.3897/zookeys.1259.169412

**Published:** 2025-11-12

**Authors:** Ya-Li Jin, Yun Bu

**Affiliations:** 1 Natural History Research Center, Shanghai Natural History Museum, Shanghai Science & Technology Museum, Shanghai, 200041, China Shanghai Natural History Museum, Shanghai Science & Technology Museum Shanghai China

**Keywords:** Genetic distance, inserted setae, Myriapoda, symphylan, taxonomy

## Abstract

The symphylans from Chongqing, Southwest China were investigated and studied for the first time. Four new species of the genus *Symphylella*, *S. obtusa***sp. nov.**, *S. yintiaolingensis***sp. nov.**, *S. flabella***sp. nov.**, and *S. micropora***sp. nov.**, are identified and described. They were compared with similar species in detail. The DNA barcodes for all new species were sequenced and analyzed together with other congeners and the genetic distance analysis further support our morphological determination. In addition, two groups, the *isabellae* group and *oligosetosa* group, of the genus *Symphylella* are proposed based on the pattern of inserted setae on the tergal processes, and their respective species are listed.

## ﻿Introduction

Symphyla is a group of common soil arthropods. However, symphylans from China are poorly studied with only 11 species recorded so far (Bu and Jin 2018; Jin and Bu 2018, 2019, 2020, 2023; Jin et al. 2019, 2023). The genus *Symphylella* Silvestri, 1902 is the most diverse group of the family Scolopendrellidae Bagnall, 1913, with 64 species described in the world (https://www.itis.gov/; Jin and Bu 2020, 2023), and six species were described in China (Jin and Bu 2023). During the comprehensive investigation of soil fauna in Jinyunshan National Nature Reserve and Yintiaoling National Nature Reserve of Chongqing, southwest China in the years 2021 and 2022, more than 400 symphylan specimens were obtained. After careful examination, four new species of the genus *Symphylella* were identified from those materials and are described in the present paper. This represents the first record of the class in Chongqing. All new species were compared with similar congeners. In addition, the DNA barcodes of the new species were sequenced and the genetic distances among *Symphylella* species were analyzed. Based on careful comparison and observation, we propose to divide the species of the genus into two morphologically wellseparated groups.

## ﻿Materials and methods

Specimens were obtained by extracting soil and litter samples from broad-leaf forests with Berlese-Tullgren funnels and then preserved in 80% ethanol. They were mounted on slides using Hoyer’s solution and dried in an oven at 50 °C. Observations were conducted under a phase contrast microscope (Leica DM 2500). Photographs were taken with a digital camera installed on the microscope (Leica DMC 4500). Line drawings were made using a drawing tube. All specimens are deposited in the collections of Shanghai Natural History Museum (**SNHM**), Shanghai, China.

The specimens used for molecular analyses were preserved in absolute ethanol at -20 °C for DNA extraction. Prior to DNA extraction, a single individual was identified to species level under a stereomicroscope. For DNA barcodes, total genomic DNA of entire individual was extracted from one specimen with Promega genomic DNA purification kit following the manufacturer’s instructions. The primer pair LCO (5’-GGTCAACAAATCATAAAGATATTGG-3’), HCO (5’-TAAACTTCAGGGTGACCAAAAAATCA-3’) (Folmer et al. 1994) was used for amplification and sequencing.

To analyze the genetic divergences among species of *Symphylella*, 14 DNA barcodes from seven species were sequenced, COI gene sequence of *Symphylella* sp. and *Scutigerella sinensis* Jin & Bu, 2023 (outgroup) were downloaded from GenBank and analyzed altogether. The detailed information and accession numbers of all sequences analyzed in this study are listed in Table 1. A Neighbor-Joining tree was constructed based on COI gene sequences by MEGA X (Kumar et al. 2018) with the Jukes-Cantor model (Jukes and Cantor 1969) and 1000 bootstrap replicates. The genetic distance (K2P-distance) was calculated using MEGA X (Kimura 1980; Kumar et al. 2018).

**Table 1.** Taxonomic and collection information of the species used in the analysis.

Species	Voucher	Locality	GenBank number	Reference
*Symphylella obtusa* sp. nov.	CQ-JYS-2021002	China: Chongqing	PX169693	present study
*Symphylella obtusa* sp. nov.	CQ-JYS-2021012	China: Chongqing	PX169694	present study
*Symphylella yintiaolingensis* sp. nov.	CQ-YTL-2022004	China: Chongqing	PX169695	present study
*Symphylella yintiaolingensis* sp. nov.	CQ-YTL-2022006	China: Chongqing	PX169696	present study
*Symphylella flabella* sp. nov.	CQ-YTL-2022007	China: Chongqing	PX169697	present study
*Symphylella micropora* sp. nov.	CQ-JYS-2022001	China: Chongqing	PX169698	present study
*Symphylella micropora* sp. nov.	CQ-YTL-2022008	China: Chongqing	PX169699	present study
*Symphylella micropora* sp. nov.	CQ-YTL-2022009	China: Chongqing	PX169700	present study
*Symphylella micropora* sp. nov.	CQ-YTL-2022010	China: Chongqing	PX169701	present study
*Symphylella macrochaeta*	SH-JZGY-2021009	China: Shanghai	PX169702	present study
*Symphylella macrochaeta*	ZJ-ZS-2020011	China: Zhejiang	PX169703	present study
*Symphylella communa*	JS-WX-2021010	China: Jiangsu	PX169704	present study
*Symphylella minuta*	JS-WX-2021008	China: Jiangsu	PX169705	present study
*Symphylella minuta*	JS-WX-2021009	China: Jiangsu	PX169706	present study
*Symphylella* sp.	YG-2006	China: Jiangsu	NC011572	Gai et al. 2008
*Scutigerella sinensis*	JYL-DJS2017011	China: Shanghai	OQ165321	Jin and Bu 2023

## ﻿Results

### ﻿Taxonomy

**Family Scolopendrellidae Bagnall, 1913**

#### ﻿Genus Symphylella

Taxon classificationAnimaliaSymphylidaScolopendrellidae

Silvestri, 1902

7A725CDD-2B8F-5CB6-BE45-50E8A169EC85

##### Type species.

*Symphylella isabellae* (Grassi, 1886); type locality: southern Italy.

#### ﻿ Symphylella obtusa sp. nov.

Taxon classificationAnimaliaSymphylidaScolopendrellidae

22E755B2-CC99-57BD-BA1B-8CF3224284A0

https://zoobank.org/167F059C-7B50-4331-B943-696F08B43B5A

**Figure 1.**
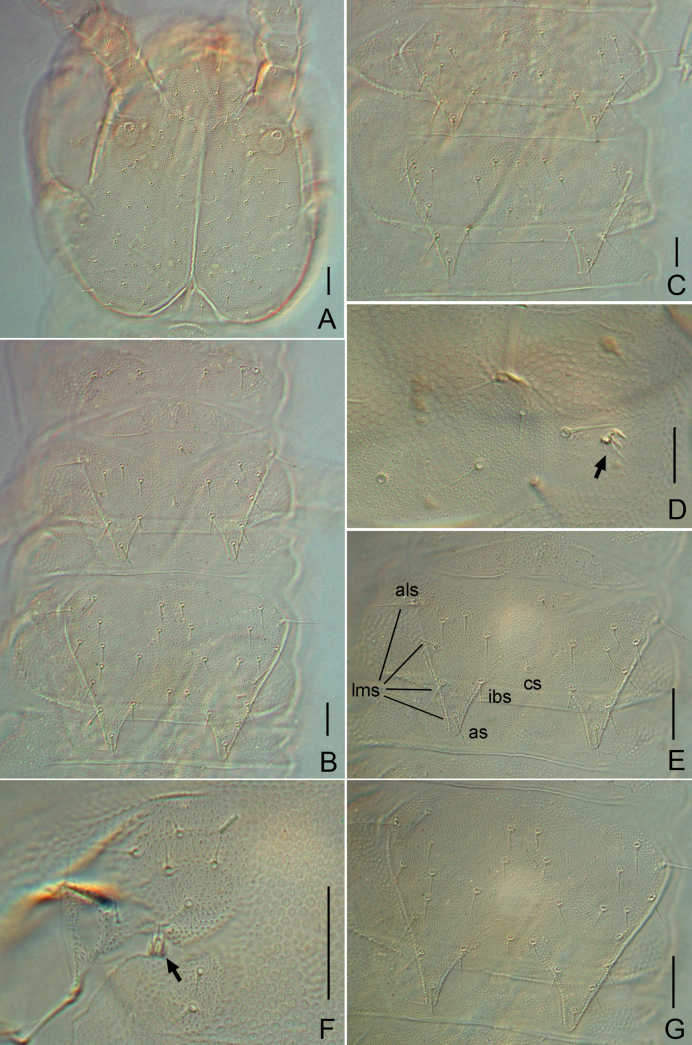
*Symphylella obtusa* sp. nov. A. Head, dorsal view; B. Tergites 1–3; C. Tergites 4 and 5; D. Leg 1, right side (arrow indicates reduced leg); E. Tergite 2; F. Left stylus and coxal sac on base of leg 11 (arrow indicates stylus); G. Tergite 3. Abbreviations: als = anterolateral seta, as = apical seta, cs = central seta, ibs = inner basal seta, lms = lateromarginal setae. Scale bars: 20 μm.

**Figure 2.**
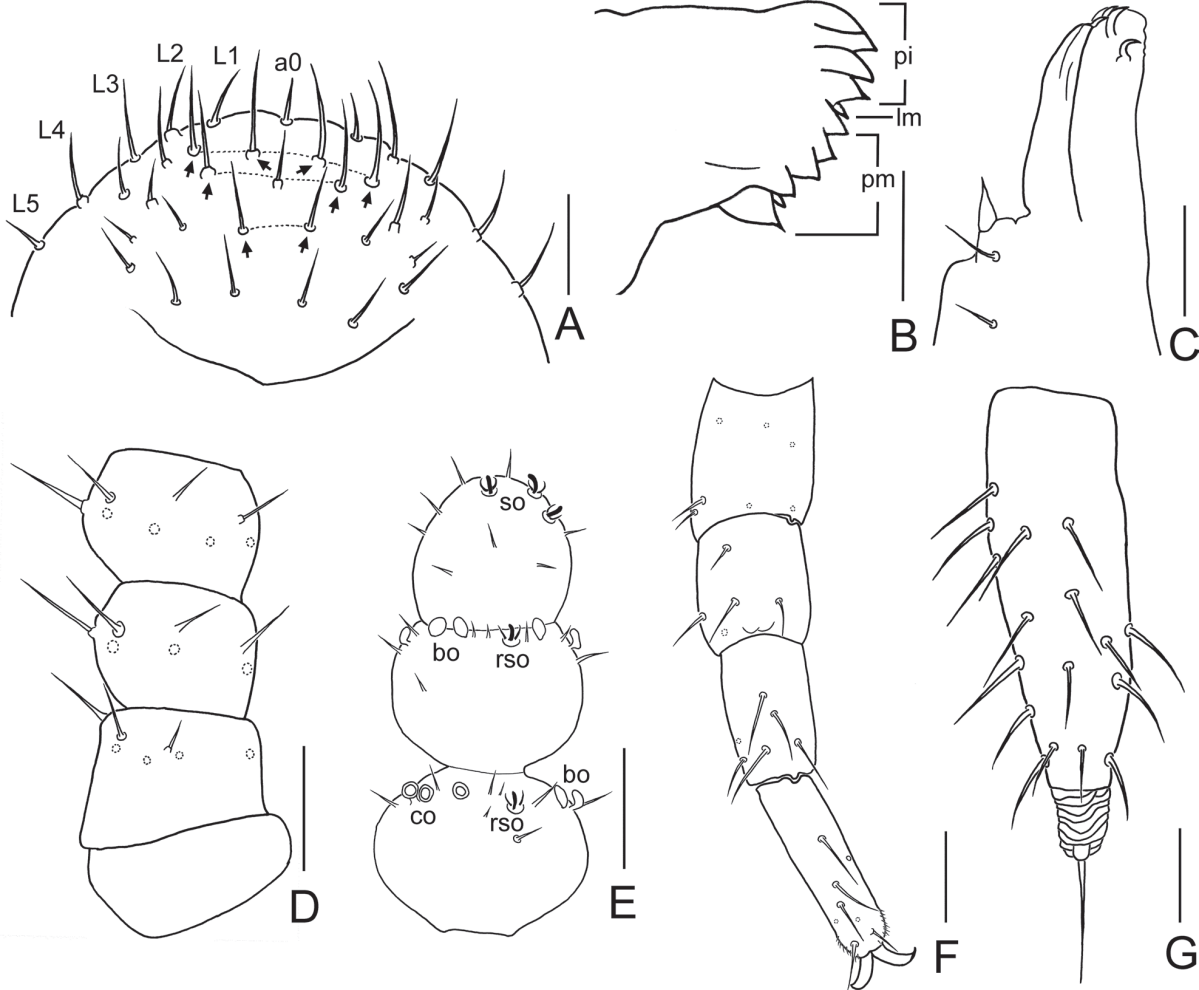
*Symphylella obtusa* sp. nov. A. Frons (arrows indicate macrosetae); B. Left mandible, lateral view; C. Left first maxilla; D. Right 1–3 antennomeres, dorsal view; E. Right terminal three antennomeres, dorsal view; F. Left leg 12, dorsal view; G. Left cercus, dorsal view. Abbreviations: a0 = antero-central seta, bo = bladder-shaped organ, co = cavity-shaped organ, L1–L5 = lateral setae, lm = lacinia mobilis, pi = pars incisivus, pm = pars molaris, rso = rudimentary spined sensory organ, so = spined sensory organ. Scale bars: 20 μm.

Figs 1, 2, Tables 2, 3, 4

##### Type material.

***Holotype***: • female (slide no. CQ-JYS-SY2021014) (SNHM), China, Chongqing, Jinyunshan National Nature Reserve, extracted from soil samples of broad-leaf forest, alt. 650 m, 29°45'N, 106°21'E, 18-X-2021, coll. Y.L. Jin, Y. Bu & S.Q. Yang. ***Paratype***: • 1 female (slide no. CQ-JYS-SY2021016) (SNHM), same data as holotype.

##### Diagnosis.

*Symphylella obtusa* sp. nov. is characterized by the 4/2/2 macrosetae on the frons, central rod divided by a weak node, 3+3 setae on the first tergite, broad processes with blunt end, inserted seta on processes absent, one and two central setae on tergites 2 and 3 respectively, long and narrow cerci. It is most similar to *S. oligosetosa* Scheller, 1971 from Peninsular India and Sri Lanka in the shapes of cerci, leg 12 and the chaetotaxy of the first tergite, but differs in the shape of central rod (divided by a weak node in *S. obtusa* sp. nov. vs not divided into two parts in *S. oligosetosa*), processes (with blunt end in *S. obtusa* sp. nov. vs with strongly pointed end in *S. oligosetosa*), and central setae of second and third tergite (respectively one and two central setae in *S. obtusa* sp. nov. vs without central seta in *S. oligosetosa*). It is also close to *S. hintoni* Edwards, 1959 from Britain in the shape of leg 12 and the chaetotaxy of the tergite, but can be easily separated by the shape of processes (broad in *S. obtusa* sp. nov. vs narrow in *S. hintoni*), and cerci (longer and narrow in *S. obtusa* sp. nov. vs short and stout in *S. hintoni*).

##### Description.

Adult body 1.5–1.7 mm long, holotype 1.7 mm.

***Head*** length 168–183 μm, width 175–188 μm, with widest part on equal level of points of articulation of mandibles. Central rod (103 μm) distinct and divided into two parts by a weak node at the middle. Anterior branches normally developed, without median branches.

Head dorsally covered with setae of different length (Fig. 1A). Frons with 5+5 lateral setae, eight macrosetae (14–22 μm) arranged as 4/2/2 (counted from anterior row to posterior row) and 2–3× as long as antero-central seta (a0) (Fig. 2A). Cuticle with fine granulation, except on anterolateral part of head with coarse granules.

***Tömösváry organ*** globular, diameter 12–13 μm, shorter than half of greatest diameter of third antennomere (28 μm), aperture small and round (7.5 μm), with striated inner wall (Fig. 1A).

***Mouthparts*.** Mandible composed of three parts: pars incisivus with four distinct thick teeth, pars molaris with four smaller teeth and two proximal spines, and lacinia mobilis with one small and pointed process observed from lateral view (Fig. 2B). First maxilla has two lobes, inner lobe with four hook-shaped teeth, palp conical and pointed (Fig. 2C). Anterior part of second maxilla with many small protuberances, each carrying one seta. Cuticle of second maxilla covered with dense pubescence.

***Antennae*** with 18–21 antennomeres (18 in holotype), ~ 0.2 of body length. First antennomere cylindrical, length ~ 0.8–1 of greatest diameter (width 23–27 μm, length 22–23 μm), with six or seven setae in one whorl, longest seta 12–13 μm. Second antennomere wider (25–28 μm) than long (19–25 μm), with seven or eight setae evenly inserted around antennal wall, longest setae 12–13 μm (Fig. 2D). Chaetotaxy of third antennomere similar to preceding ones (Fig. 2D). Setae on proximal antennomeres longer and on distal antennomeres shorter. Proximal antennomeres with only primary whorl of setae (Fig. 2D). Secondary whorl appearing ventrally on antennomeres 11. Four kinds of sensory organs observed on antenna (Fig. 2E): rudimentary spined sensory organs (*ros*) on dorsal side of subterminal antennomeres; spined sensory organs (*so*) consisting of three or four curved spines around one central pillar, only present on apical antennomere; cavity-shaped organs (*co*) on dorsal side of antennomeres 6 or 7 to penultimate; bladder-shaped organs (*bo*) on antennomeres 13–16 to penultimate increasing in number to maximum 15 . Apical antennomere sub-spherical, with its length as long as width (21–27 μm), with five *so* and 13–15 setae located distally (Fig. 2E). All antennomeres covered with short pubescence. Chaetotaxy and sensory organs on antennae of holotype are given in Table 2.

**Table 2.** Numbers of setae and sensory organs on antennae of *Symphylella obtusa* sp. nov. (holotype, excluding apical antennomere).

Antennomere	Primary whorl setae	Secondary whorl setae	Rudimentary spined sensory organs	Cavity-shaped organs on dorsal side	Bladder-shaped organs
1	6				
2	8				
3	9				
4	8				
5	8				
6	8			1	
7	8			1	
8	8			1	
9	10			1	
10	10			1	
11	10	1			
12	10	1		1	
13	10	2			1
14	10	2		1	1
15	11	3			4
16	11	2	1	3	10
17	11	4	1		12

***Trunk*** with 17 tergites. Tergites 2–13, and 15 each with one pair of triangular processes. Length from base to tip of processes somewhat longer than or same as its basal width; basal distance between processes distinctly longer than their length from base to tip (Table 3). All processes without end-swellings (Fig. 1B, C, E, G). Definition of chaetotaxy on tergite as follows: anterolateral setae (*als*) located on anterolateral angle of each tergite; apical seta (*as*) close to process apex; lateromarginal setae (*lms*) located on lateral margin of process and including *als* and *as*; inner basal setae (*ibs*) located on inner base of processes; inserted setae (*is*) present between *ibs* and *as*; central setae (*cs*) present at base of processes between *ibs*; other setae including all setae except above nominated ones. Anterolateral setae of tergites 2, 3, 4, 6, 7, 9, 10, and 12 distinctly longer than other *lms* of same tergite, those of tergites 5, 8, 11, 13 and 15 subequal or slightly shorter than longest ones of other *lms.* All processes without *is* seta. All tergites pubescent (Fig. 1B, C, E, G).

**Table 3.** Chaetotaxy of tergites of *Symphylella obtusa* sp. nov. (holotype in brackets, left side/ right side).

Tergite	Lateromarginal setae	Central setae	Other setae
1	3+3		
2	5 (5)	1 (1)	7–8 (7)
3	5–6 (5/6)	2 (2)	14 (14)
4	4(4)	2 (2)	6–7 (7)
5	4–5 (5/4)	2–3 (2)	6–7 (7)
6	6–7 (7/6)	2–3 (3)	14–17 (14)
7	3–4 (4/3)	2–3 (3)	7–9 (7)
8	5 (5)	3 (3)	5–9 (5)
9	6–7 (6/7)	3 (3)	13–17 (13)
10	4 (4)	1–3 (3)	7 (7)
11	4–5 (5/4)	2–3 (2)	6–7 (6)
12	6 (6)	2–4 (2)	16–17 (16)
13	4–5 (4/5)	2–3 (3)	7–8 (8)
14			17–20 (20)
15	4–5 (4)	2 (2)	11–12 (12)
16			10–11 (11)
17			14–15 (14)

***Tergites*.** Tergite 1 reduced, with 3+3 setae of different lengths (Fig. 1B). Tergite 2 complete, with two triangular posterior processes, four or five *lms*, one *cs*, with *als* 0.7–0.8 as long as length of process, length of processes almost as long as broad, basal distance between processes 1.4–1.6× as long as their length (Fig. 1B, E). Tergite 3 complete, broader and longer than preceding one with ratios of 0.8–0.9, 0.9–1.0, and 1.5 respectively, 5–6 *lms*, two *cs* (Fig. 1B, G). Tergite 4 broader than tergite 3, with ratios 0.9–1.3, 0.8–0.9 and 1.8–2.5 respectively, four *lms*, two *cs* (Fig. 1C). Chaetotaxy of tergites 5–7, 8–10, and 11–13 similar to tergites 2–4 (Fig. 1C). Pattern of alternating tergite lengths of two short tergites followed by a long tergite only disrupted at caudal end (Table 3). Tergites 14, 16, and 17 without processes, with 17–20, 10 or 11, and 14 or 15 setae, respectively. Chaetotaxy and measurements of tergites are given in Tables 3, 4.

**Table 4.** Measurements of tergites and processes of *Symphylella obtusa* sp. nov. (holotype in brackets, in μm).

Tergite	Length	Width	Length of processes	Basal width of processes	Basal distance between processes
1	20–25 (25)	100 (100)			
2	38–40 (38)	100–105 (100)	22–25 (22)	23–26 (23)	35–36 (35)
3	60–65 (65)	128–132 (128)	24–26 (24)	27–28 (28)	35–40 (35)
4	40–45 (45)	140 (140)	20–25 (25)	23–30 (30)	46–51 (46)
5	43–48 (48)	122–125 (122)	24–28 (28)	25 (25)	48–50 (48)
6	80–90 (90)	160–170 (170)	26–27 (27)	26–30 (30)	55–58 (55)
7	45–56 (56)	162–163 (162)	20–25 (25)	21–23 (23)	67–68 (68)
8	45–55 (55)	146–148 (146)	23–29 (29)	21–26 (26)	60–65 (60)
9	80–85 (85)	188–192 (188)	26–28 (28)	23–31 (31)	62–66 (62)
10	40–55 (55)	170–180 (180)	21–26 (26)	21–28 (28)	72–80 (72)
11	45–52 (52)	145–155 (155)	23–26 (26)	20–23 (23)	69–70 (70)
12	78–85 (78)	136–182 (136)	25–29 (29)	24 (24)	65–69 (65)
13	50–55 (55)	164–172 (172)	19–20 (20)	23–24 (23)	65–68 (68)
14	50–60 (50)	145–146 (145)			
15	66–68 (66)	155–162 (162)	16–19 (19)	19–20 (19)	52–55 (52)
16	50 (50)	112–123 (123)			
17	70–80 (80)	100–108 (108)			

***Legs*.** First pair of legs reduced to two small hairy cupules, each with one long seta (9 μm) (Fig. 1D). Basal areas of legs 2–12 each with 3–6 setae (Fig. 1F). Leg 12 somewhat shorter than length of head (Fig. 2F), trochanter 1.1–1.5× as long as wide (33–47 μm, 30–31 μm), with 6–9 subequal setae; femur almost as long as wide (27–28 μm, 26–27 μm), with five setae, four on dorsal or outer side, one on ventral side, longest dorsal setae (11–12 μm) ~ 0.4 of greatest diameter of podomere; tibia ~1.7–1.9× longer than wide (35–38 μm, 20–21 μm), with five dorsal setae and one ventral seta, longest dorsal seta (13 μm) ~ 0.6 of greatest diameter of tibia; tarsus cylindrical, ~ 3.3–3.5× as long as wide (35–38 μm, 20–21 μm) with six dorsal setae, two of them close to claw, longest dorsal seta (12–13 μm) slightly shorter than greatest width of tarsus, two ventral setae close to claw distinctly shorter than dorsal one. Claws curved, anterior one somewhat broader than posterior one, the latter more curved than former (Fig. 2F). All legs covered with dense pubescence.

***Coxal sacs*** present at bases of legs 3–9, fully developed, each with four or five setae on surface. Corresponding areas of legs 2, 10, 11, and 12 replaced by one or two setae (Fig. 1F).

***Styli*** present at base of legs 3–12, short and sub-cylindrical (length 3–4 μm, width 3 μm), basal part with dense straight hairs; distal hairless, with an apical hair (2 μm) (Fig. 1F).

***Sense calicles*** located on two ventral protuberances of last tergite, posterior to base of leg 12, with smooth margin around pit. Sensory seta inserted in cup center, extremely long (138–163 μm).

***Cerci*** narrow and tapered, 3.9–4.3× as long as its greatest width (90–98 μm, 21–25 μm), sparsely covered with 25 or 26 setae (Fig. 2G). Proximal setae somewhat longer than distal ones. Four or five long and straight setae located on dorsal and outer side, other setae slightly curved. Longest outer seta (17–18 μm) straight and slightly longer than greatest width of cerci. Terminal area short (18 μm), 0.7–0.9 as long as greatest width of cerci, circled by several layers of curved ridges. Terminal seta (23–25 μm) longer than terminal area (Fig. 2G).

##### Etymology.

The species name *obtusa* derived from the Latin word *obtus* means blunt, referring to the blunt end of the process on the tergite.

##### Distribution.

China (Chongqing).

#### ﻿ Symphylella yintiaolingensis sp. nov.

Taxon classificationAnimaliaSymphylidaScolopendrellidae

E832C56F-0117-590C-907C-4D5DC56E686E

https://zoobank.org/1B4CF55A-537F-491D-B6AF-03908EE6C1DE

**Figure 3.**
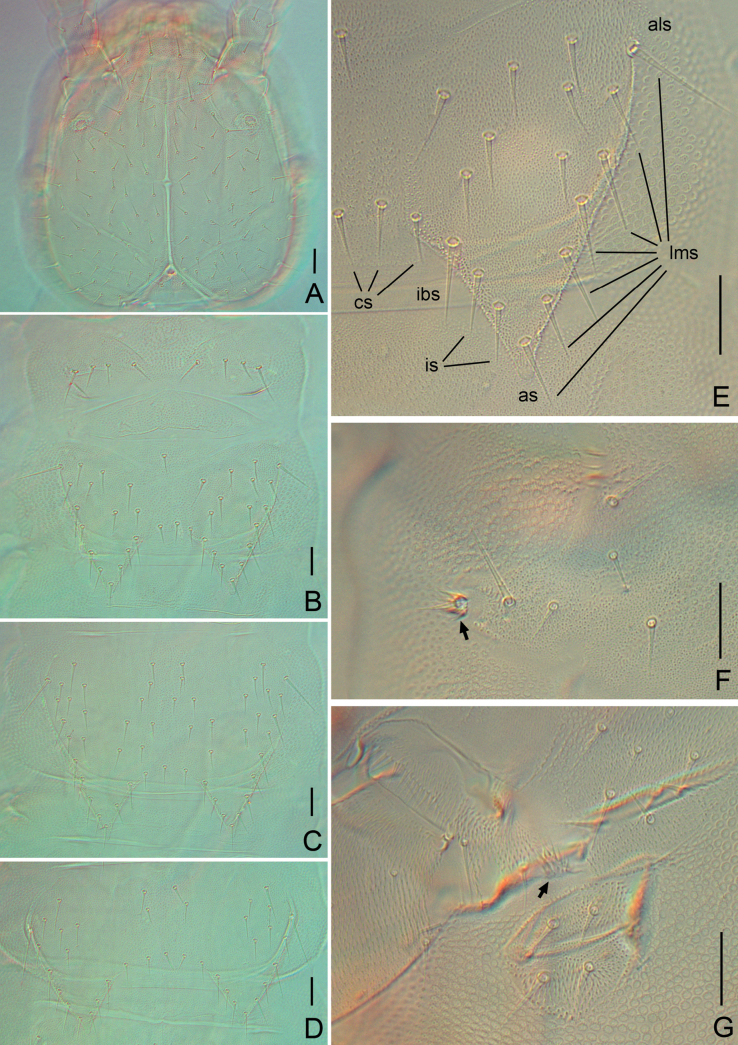
*Symphylella yintiaolingensis* sp. nov. A. Head, dorsal view; B. Tergites 1 and 2; C. Tergite 3; D. Tergite 4; E. Tergite 2; F. Leg 1, left side (arrow indicates reduced leg); G. Left stylus and coxal sac on base of leg 5 (arrow indicates stylus). Abbreviations: als = anterolateral seta, as = apical seta, cs = central seta, ibs = inner basal seta, is = inserted setae, lms = lateromarginal setae. Scale bars: 20 μm.

**Figure 4.**
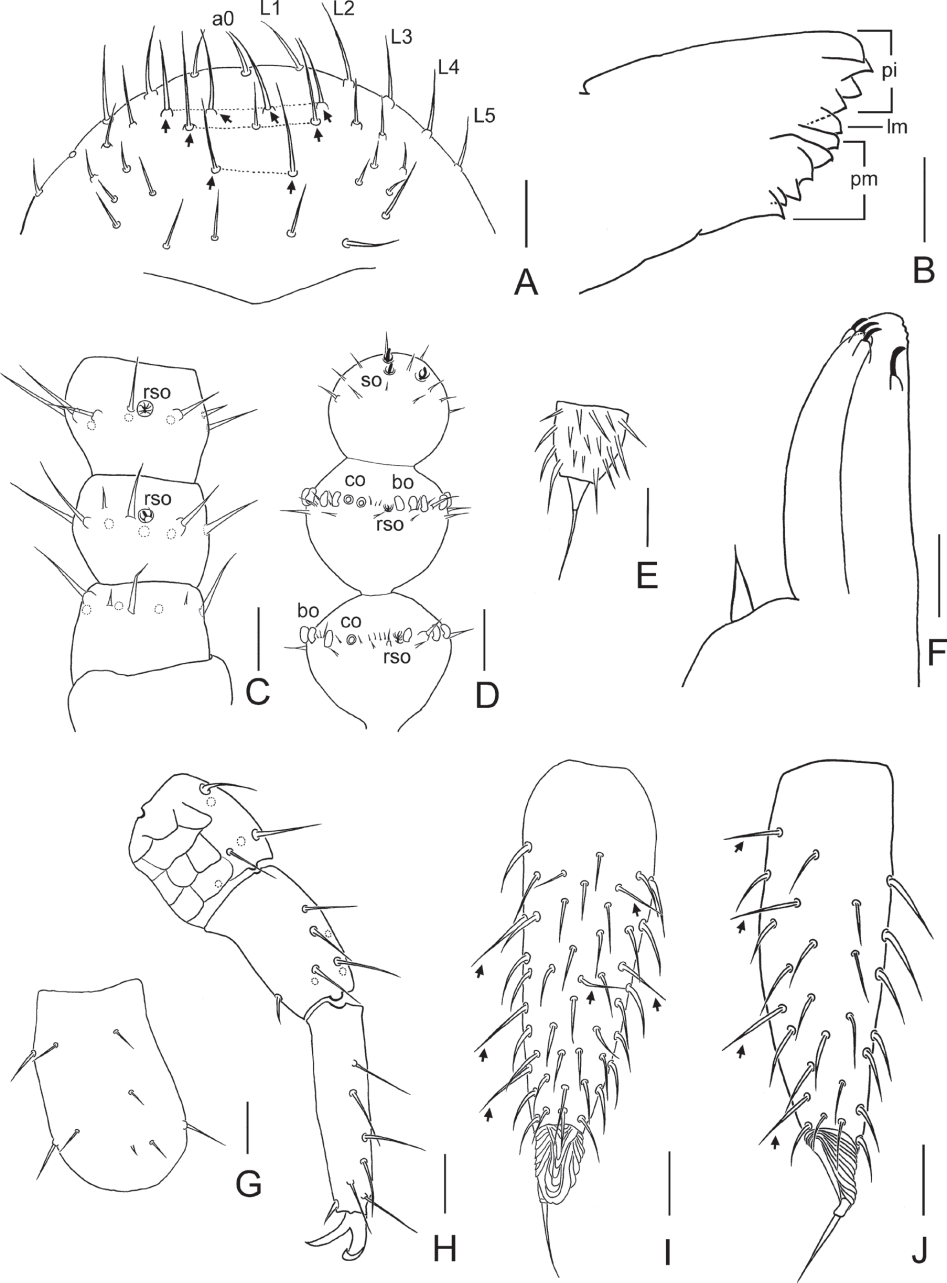
*Symphylella yintiaolingensis* sp. nov. A. Frons (arrows indicate macrosetae); B. Left mandible, lateral view; C. Right 1–3 antennomeres, dorsal view; D. Right terminal three antennomeres, dorsal view; E. Left stylus at base of leg 5; F. Left first maxilla; G. Trochanter of leg 12, right, dorsal view; H. Femur, tibia and tarsus of leg 12, right, dorsal view; I. Left cercus, ventral view (arrows indicate long and erect setae); J. Left cercus, lateral view (arrows indicate long and erect setae on ventral side) . Abbreviations: a0 = antero-central seta, bo = bladder-shaped organ, co = cavity-shaped organ, L1–L5 = lateral setae, lm = lacinia mobilis, pi = pars incisivus, pm = pars molaris, rso = rudimentary spined sensory organ, so = spined sensory organ. Scale bars: 20 μm (A–D, F–J); 5 μm (E).

Figs 3, 4, Tables 5, 6, 7

##### Type material.

***Holotype***: • female (slide no. CQ-YTL-SY2022013) (SNHM), China, Chongqing Municipality, Wuxi County, Yintiaoling National Natural Reserve, Tianchiba, extracted from soil samples of broad-leaf forest, alt. 2100 m, 31°28'N, 109°47'E, 12-VIII-2022, coll. Y. Bu & Y. L. Jin. ***Paratypes***: • 1 male (slide no. CQ-YTL-SY2022014) (SNHM), same data as holotype; 2 females (slides no. CQ-YTL-SY2022015, CQ-YTL-SY2022016) (SNHM), same data as holotype; • 1 female (slide no. CQ-YTL-SY2022025) (SNHM), China, Chongqing Municipality, Wuxi County, Yintiaoling National Natural Reserve, Linkouzi, extracted from soil samples of broad-leaf forest, alt.1595 m, 31°28'N, 109°52'E, 14-VIII-2022, coll. Y. Bu & Y. L. Jin; • 4 females (slides no. CQ-YTL-SY2022030–CQ-YTL-SY2022033) (SNHM), China, Chongqing Municipality, Wuxi County, Yintiaoling National Natural Reserve, Linkouzi, Zhuanping, extracted from soil samples of broad-leaf forest, alt. 1250 m, 31°29'N, 109°54'E, 15-VIII-2022, coll. Y. Bu & Y. L. Jin.

##### Diagnosis.

*Symphylella yintiaolingensis* sp. nov. is characterized by 4+4 setae arranged in two groups on the first tergite, 1–3 inserted setae on processes, moderately swollen ends of processes, pointed apical seta of the stylus, long and erect setae present on the outer and ventral sides of cerci. It belongs to the group of species with inserted setae present on the process of the tergite. It is most similar to *S. communa* Jin & Bu, 2020 from East China in the chaetotaxy of tergites, the shapes of the styli and the cercus, but differs in the shape of the Tömösváry organ (with larger aperture in *S. yintiaolingensis* sp. nov. vs smaller aperture in *S. communa*), and in the shape of the stylus (with pointed apical seta in *S. yintiaolingensis* sp. nov. vs blunt apical seta in *S. communa*). The new species is also similar to the widespread *S. vulgaris* Hansen, 1903 in the shape of the tergites, but they can be easily separated by the chaetotaxy of first tergite (4+4 setae in *S. yintiaolingensis* sp. nov. vs 3+3 setae in *S. vulgaris*), the erect setae on cercus (present on outer and ventral sides in *S. yintiaolingensis* sp. nov. vs present only on ventral side in *S. vulgaris*).

##### Description.

Adult body 2.4 mm long on average (2.0–2.7 mm, *n* = 9), holotype 2.6 mm.

***Head*** as long as wide, length 230–270 μm, width 190–225 μm, with widest part on equal level as points of articulation of mandibles. Central rod well developed, divided into two portions by a node-like sub-middle interruption, with anterior part 60–75 μm and posterior part 63–75 μm. Dorsal side of head moderately covered with setae of different length (Fig. 3A). Frons with 5+5 lateral setae, macrosetae (21–30 μm) arranged as 4/2/2 and 2.0–2.5× as long as antero-central seta (a0), and 18 normal setae (Fig. 4A). Cuticle on anterolateral part of head with coarse granules (Fig. 3A).

***Tömösváry organ*** globular, diameter 15–20 μm, almost half of greatest diameter of third antennomere (30–32 μm), aperture round (8–13 μm), with distinct vertical inner striae (Fig. 3A).

***Mouthparts*.** Mandible composed of three parts: pars incisivus (*pi*) with four distinct thick teeth, pars molaris (*pm*) with four teeth and two proximal spines, and lacinia mobilis (*lm*) with one blunt process observed from lateral view (Fig. 4B). First maxilla with two lobes, inner lobe with four hook-shaped teeth, palp pointed and sharp (Fig. 4F). Anterior part of second maxilla with many small protuberances, each carrying one seta, distal setae thick; posterior part with sparse setae. Cuticle of second maxilla covered with dense pubescence.

***Antennae*** with 14–19 antennomeres (16 in holotype), ~ 0.2 of body length. First antennomere cylindrical, 1.3–1.6× as wide as long (width 35–42 μm, length 24–30 μm), with 5–6 setae in one whorl, one minute seta on dorsal side of antennomere. Second antennomere wider (35–43 μm) than long (29–32 μm), with 8 setae inserted around antennal wall, interior setae (21–22 μm) longer than exterior ones (17–18 μm). Chaetotaxy of third antennomere similar to preceding ones (Fig. 4C). Setae on proximal antennomeres longer and on distal antennomeres shorter. Proximal antennomeres with only primary whorl of setae, in middle and subapical antennomeres with several minute setae in secondary whorl. Four kinds of sensory organs observed on antenna (Fig. 4D): *rso* on dorsal side from second to subterminal antennomeres; *so* only present on apical antennomere; *co* on antennomeres 5 and 6 to apical one (absent on apical antennomere in some specimens), increasing in number to maximum three on subterminal antennomere; *bo* irregular, oblate or curved, present on antennomeres 5–7 to penultimate, increasing in number to maximum 22 on penultimate antennomere. Apical antennomere sub-spherical, somewhat wider than long (width 30–32 μm, length 27–28 μm), with five *so* and 15–18 short setae apically (Fig. 4D). All antennomeres covered with short pubescence. Chaetotaxy and sensory organs of antennae of holotype are given in Table 5.

**Table 5.** Numbers of setae and sensory organs on antennae of *Symphylella yintiaolingensis* sp. nov. (holotype, excluding apical antennomere).

Antennomere number	Primary whorl setae	Secondary whorl setae	Rudimentary spined sensory organs	Cavity-shaped organs on dorsal side	Bladder-shaped organs
1	6				
2	8		1		
3	9		1		
4	10		1		
5	11		1		
6	10	1		1	
7	11	3	1	1	1
8	12	4	1	1	1
9	12	7	1	1	1
10	13	7	1	1	1
11	14	7	1	1	3
12	14	5	1	1	4
13	13	4		1	4
14	13	5		1	6
15	12	4		1	11
16	10	5		1	18
17	10	6		3	18

***Trunk*.** Length from base to tip of triangular processes somewhat shorter than or the same as its basal width; basal distance between processes of tergites distinctly longer than their length from base to tip except for tergites 2 and 3, in which basal distances shorter than lengths of processes (Table 6). All processes with moderate swollen ends. Anterolateral setae of tergites 2, 3, 4, 6, 7, 9, 10, and 12 distinctly longer than other *lms* of same tergite, those of tergites 5, 8, 11, 13, and 15 subequal or slightly shorter than longest ones of other *lms.* Anterolateral setae of tergites shorter than or subequal to process of same tergite. Processes with 1–3 *is.* All tergites pubescent (Fig. 3B–E).

**Table 6.** Chaetotaxy of tergites of *Symphylella yintiaolingensis* sp. nov. (holotype in brackets).

Tergite	Lateromarginal setae	Inserted seta	Central setae	Other setae
1	4+4 or 3+4 (4+4)			
2	6–7 (7)	1–2 (2)	1–3 (3)	7–14 (13)
3	8–9 (9)	1–3 (1–2)	2–4 (3)	18–26 (26)
4	5–7 (6)	1–2 (2)	3–5 (5)	10–16 (13)
5	5–7 (5)	1–3 (2)	4 (4)	11–17 (17)
6	8–10 (9)	1–3 (2)	3–5 (4)	23–39 (32)
7	5–8 (5)	1–2 (1–2)	4–6 (6)	12–18 (18)
8	5–8 (7–8)	1–2 (2)	3–6 (5)	12–18 (15)
9	8–11 (9)	1–2 (2)	4–6 (5)	23–36 (30)
10	4–9 (6–7)	1 (1)	4–7 (5)	8–19 (19)
11	5–7 (6–7)	1 (1)	3–6 (6)	8–18 (17)
12	7–10 (9–10)	1–2 (1–2)	3–6 (4)	21–33 (28)
13	5–6 (6)	0–2 (1)	3–7 (6)	8–15 (13)
14				16–31 (26)
15	5–8 (6–7)	0–2 (1)	0–5 (4)	11–26 (25)
16				11–21 (16)
17				16–34 (29)

***Tergites*.** Tergite 1 reduced, with 4+4 subequal setae, asymmetrically lacking one seta in three paratypes (Fig. 3B). Tergite 2 complete, with two triangular posterior processes, 6–7 *lms*, one or two *is*, 1–3 *cs*, *als* 0.8–0.9 of length of process, processes 0.8–1.0 time as long as broad, basal distance between processes 0.6–0.9 as long as their length (Fig. 3B, E). Tergite 3 complete, broader, and longer than preceding one with ratios of 0.8–1.2, 0.8–1.0 and 0.8–1.0 respectively, 8–9 *lms*, 1–3 *is*, 2–4 *cs* (Fig. 3C). Tergite 4 broader than tergite 3, with ratios 0.9–1.2, 0.6–0.7 and 1.7–2.6 respectively, 5–7 *lms*, one or two *is*, 3–5 *cs.* Chaetotaxy of tergites 5–7, 8–10 and 11–13 similar to tergites 2–4 (Fig. 3D). Pattern of alternating tergite lengths of two short tergites followed by one long tergite only disrupted at caudal end. Tergites 14, 16, and 17 without processes, with 16–31, 11–21, and 16–34 setae, respectively. Chaetotaxy and measurements of tergites are given in Tables 6, 7.

**Table 7.** Measurements of tergites and processes of *Symphylella yintiaolingensis* sp. nov. (holotype in parentheses, in μm).

**Tergite**	**Length**	**Width**	**Length of processes**	**Basal width of processes**	**Basal distance between processes**
1	40 (40)	150 (150)			
2	50–75 (58)	150–170 (155)	36–45 (40)	40–50 (40)	25–38 (38)
3	93–125 (110)	175–210 (200)	40–47 (45)	45–55 (48)	25–50 (43)
4	50–83 (70)	200–250 (220)	30–40 (35)	45–65 (53)	63–83 (70)
5	63–88 (88)	198–225 (213)	40–50 (47)	43–55 (48)	63–80 (73)
6	112.5–150 (130)	243–290 (280)	42–55 (55)	48–60 (58)	63–85 (68)
7	58–95 (83)	233–295 (275)	35–47 (41)	47–70 (58)	85–110 (100)
8	63–98 (75)	213–270 (250)	42–50 (50)	50–57 (52)	78–105 (95)
9	108–150 (125)	263–315 (313)	45–52 (52)	47–63 (52)	70–108 (95)
10	60–95 (75)	248–330 (288)	32–42 (37)	47–62 (52)	88–120 (113)
11	75–90 (75)	225–278 (270)	37–47 (45)	50–60 (55)	90–115 (105)
12	100–163 (120)	258–325 (313)	37–47 (47)	53–60 (60)	75–118 (98)
13	43–88 (43)	230–325 (285)	25–42 (42)	50–62 (60)	88–125 (118)
14	58–8 (65)	215–260 (250)			
15	83–115 (108)	225–313 (270)	23–40 (30)	50–63 (56)	55–85 (76)
16	50–70 (63)	20–228 (20)			
17	113–125 (125)	150–188 (175)			

***Legs*.** First pair of legs reduced to two small hairy cupules, each with one long seta (10–11 μm) (Fig. 3F). Basal areas of legs 2–12 each with 5–11 setae (Fig. 3G). Leg 12 as long as head length, trochanter 1.1–1.6× longer than wide (64–80 μm, 45–60 μm), with 6–9 subequal setae in total (Fig. 4G); femur almost as long as wide (38–50 μm, 35–50 μm), with five or six setae, longest dorsal seta (18–23 μm) 0.5–0.7 of greatest diameter of podomere in length, pubescent dorsally, laterally with cuticular thickenings in pattern of scales; tibia nearly 1.4–1.9× longer than wide (45–58 μm, 28–35 μm), with 6–8 setae, longest dorsal one (20–25 μm) 0.7–0.9 of greatest diameter of tibia; tarsus sub-cylindrical, 3.2–5.4× as long as wide (63–95 μm,18–20 μm) with six dorsal setae: four straight and protruding, two depressed, longest seta (15–22 μm) almost as long as greatest diameter of tarsus, one ventral setae close to claw and distinctly shorter than dorsal ones. Claws slightly curved, anterior one broader and more curved than posterior one (Fig. 4H). All legs covered with dense pubescence except areas with cuticular thickenings.

***Coxal sacs*** present at bases of legs 3–9, fully developed, each with four or five setae on surface (Fig. 3G). Corresponding areas of legs 2, 10, 11, and 12 replaced by 1–4 setae, respectively.

***Styli*** present at base of legs 3–12, short and sub-conical (length 8 μm, width 5 μm), basal part with straight hairs; distal quarter hairless, with an apical seta (2 μm) (Figs 3G, 4E).

***Sense calicles*** with smooth margin around pit. Sensory seta inserted in cup center, extremely long (138–200 μm).

***Cerci*** ~ 0.7 of head in length, 2.9–3.5× as long as its greatest width (150–183 μm, 43–63 μm), densely covered with 65–96 setae (Fig. 4I). Two types of setae inserted on cercus: several long and erect setae located on outer and ventral side, other setae slightly curved and depressed. Longest outer long and erect seta (24–28 μm) ~ 1/2 of greatest width of cerci, terminal area short (24–35 μm), circled by 7–9 layers of curved ridges. Terminal seta (25–33 μm) close in length with terminal area (Fig. 4I, J).

##### Etymology.

The new species is named after the type locality Yingtiaoling National Nature Reserve.

##### Distribution.

China (Chongqing).

#### ﻿ Symphylella flabella sp. nov.

Taxon classificationAnimaliaSymphylidaScolopendrellidae

17F38253-FAC5-5BD8-ADB0-82A4979A913F

https://zoobank.org/EBFEE4A1-CC35-4C3B-9CFE-F23136CE0AAE

**Figure 5.**
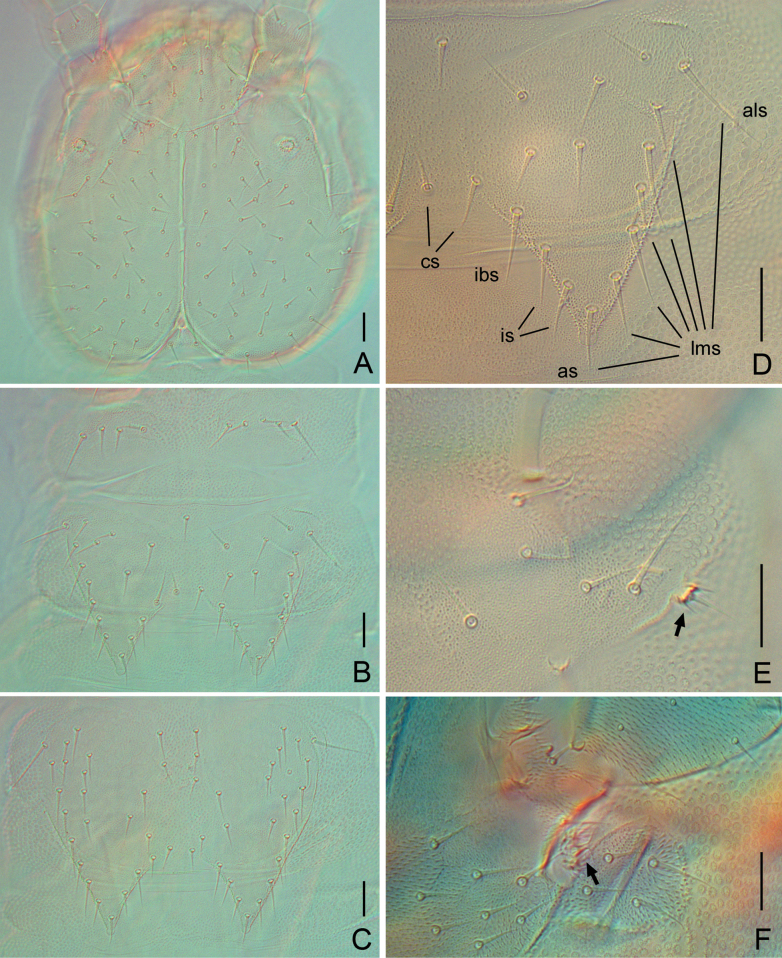
*Symphylella flabella* sp. nov. A. Head, dorsal view; B. Tergites 1 and 2; C. Tergite 3; D. Tergite 2; E. Leg 1, right side (arrow indicates reduced leg); F. Right stylus and coxal sac on base of leg 6 (arrow indicates stylus). Abbreviations: als = anterolateral seta, as = apical seta, cs = central seta, ibs = inner basal seta, is = inserted setae, lms = lateromarginal setae. Scale bars: 20 μm.

**Figure 6.**
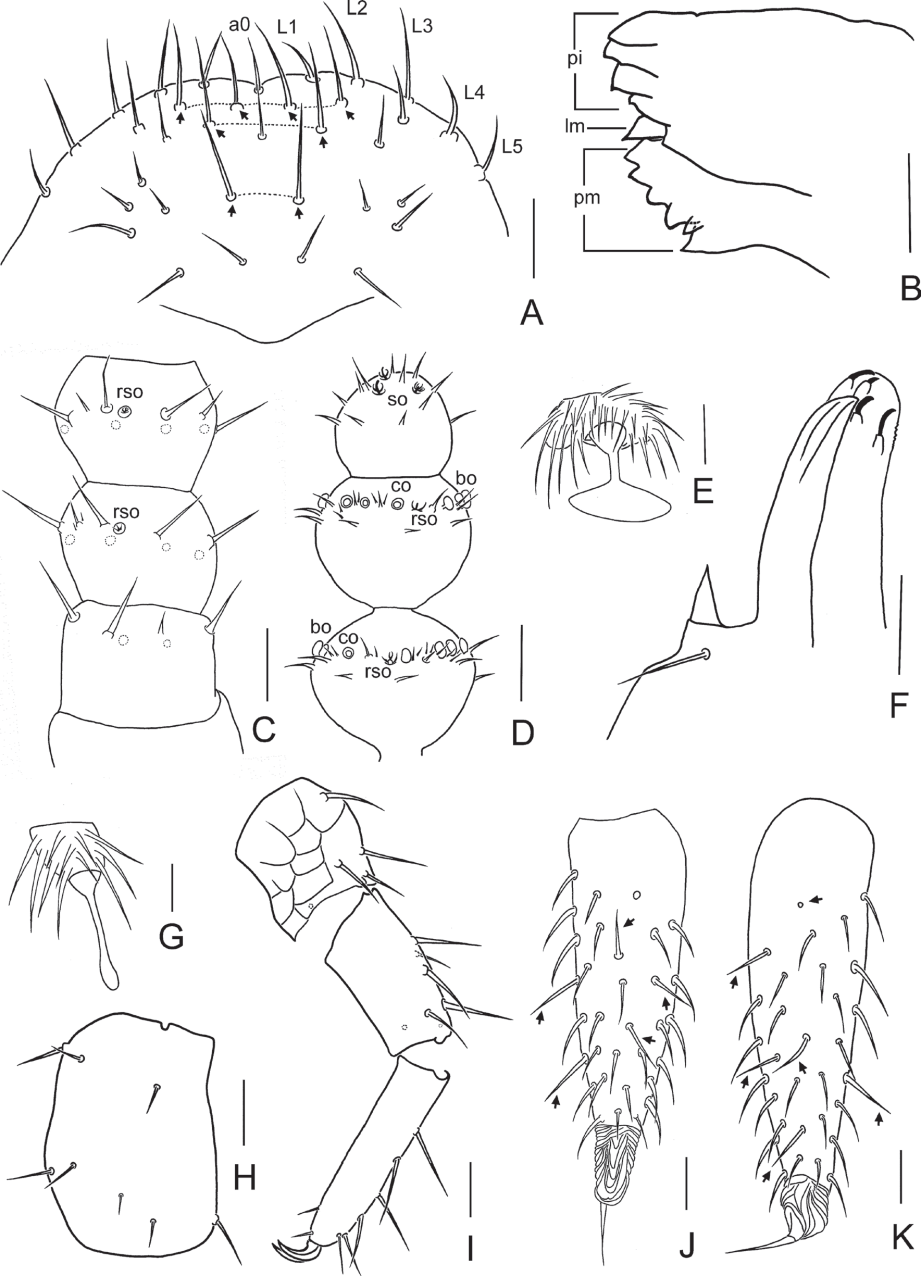
*Symphylella flabella* sp. nov. A. Frons (arrows indicate macrosetae); B. Right mandible, lateral view; C. Right 1–3 antennomeres, dorsal view; D. Right terminal three antennomeres, dorsal view; E. Right stylus at base of leg 6; F. Left first maxilla; G. Left stylus at base of leg 12; H. Trochanter of leg 12, right, ventral view; I. Femur, tibia and tarsus of leg 12, right, dorsal view; J. Left cercus, ventral view (arrows indicate long and erect setae); K. Right cercus, lateral view (arrows indicate long and erect setae). Abbreviations: a0 = antero-central seta, bo = bladder-shaped organ, co = cavity-shaped organ, L1–L5 = lateral setae, lm = lacinia mobilis, pi = pars incisivus, pm = pars molaris, rso = rudimentary spined sensory organ, so = spined sensory organ. Scale bars: 20 μm (A–D, F, H–K); 5 μm (E, G).

Figs 5, 6, Tables 8, 9, 10

##### Type material.

***Holotype***: • female (slide no. CQ-YTL-SY2022026) (SNHM), China, Chongqing Municipality, Wuxi County, Yintiaoling National Natural Reserve, Linkouzi, alt. 1250 m, 31°28'N, 109°52'E, 14-VIII-2022, coll. Y. Bu & Y. L. Jin. ***Paratypes***: • 1 female (slide no. CQ-YTL-SY2022023) (SNHM), same data as holotype; • 1 female (slide no. CQ-YTL-SY2022029) (SNHM), China, Chongqing Municipality, Wuxi County, Yintiaoling National Natural Reserve, Linkouzi, Zhuanping, extracted from soil samples of broad-leaf forest, alt. 1595 m, 31°29'N, 109°54'E, 15-VIII-2022, coll. Y. Bu & Y. L. Jin.

##### Diagnosis.

*Symphylella flabella* sp. nov. is characterized by distinct roundish swollen ends of processes, 1–3 inserted setae on processes, fan-shaped apex of styli at base of legs 3–10, sub-conical basically and with blunt, slightly swollen apex in legs 11 and 12, long and erect setae present on outer and ventral sides of cerci. It is affiliated to *S. yintiaolingensis* in the chaetotaxy of tergites, the shape of the Tömösváry organ, leg 12, and cercus, but can be easily distinguished by the shape of styli (with fan-shaped apex at base of legs 3–10 in *S. flabella* sp. nov. vs all styli with pointed apices in *S. yintiaolingensis* sp. nov.) and by the shape of processes (with distinct rounded swollen end in *S. flabella* sp. nov. vs moderately swollen end in *S. yintiaolingensis* sp. nov.).

##### Description.

Adult body 2.5 mm long on average (2.4–2.6 mm, *n* = 3), holotype 2.5 mm.

***Head*** as long as wide, length 230–240 μm, width 220–230 μm, with widest part at level of points of articulation of mandibles. Central rod well developed, divided into two portions by node-like sub-middle interruption, both anterior and posterior parts 60–63 μm. Dorsal side of head moderately covered with setae of different length (Fig. 5A). Frons with 5+5 lateral setae, eight macrosetae (22–28 μm) arranged as 4/2/2 and 1.5× as long as antero-central seta (a0), and 15–16 other setae (Fig. 6A). Cuticle on anterolateral part of head with coarse granules (Fig. 5A).

***Tömösváry organ*** globular, diameter 14–16 μm, shorter than half of greatest diameter of third antennomere (38–40 μm), aperture round, 8–9 μm (Fig. 5A).

***Mouthparts*.** Mandible composed of three parts: pars incisivus (*pi*) with four distinct thick teeth, pars molaris (*pm*) with four smaller teeth and two proximal spines, and lacinia mobilis (*lm*) with one sharp process observed from lateral view. (Fig. 6B). First maxilla with two lobes, inner lobe with four hook-shaped teeth, palp of first maxilla sharp (Fig. 6F). Anterior part of second maxilla with many small protuberances, each carrying one seta, distal setae thick; posterior part with sparse setae. Cuticle of second maxilla covered with dense pubescence.

***Antennae*** with 17–19 antennomeres (17 in holotype), ~ 0.2 of body length. First antennomere cylindrical, 1.4–1.7× as wide as long (width 36–40 μm, length 23–26 μm), with 5–6 setae in one whorl, one minute seta on dorsal side of antennomere (Fig. 6C). Second antennomere wider (36–40 μm) than long (28–30 μm), with 8–9 setae inserted around antennal wall, interior setae (20–22 μm) longer than exterior ones (17–18 μm). Chaetotaxy of third antennomere similar to preceding ones (Fig. 6C). Setae on proximal antennomeres longer and on distal antennomeres shorter (Fig. 6C, D). Proximal antennomeres with only primary whorl of setae, in middle and subapical antennomeres with several minute setae in secondary whorl. Four types of sensory organs observed on antennae (Fig. 6D): *rso* on dorsal side of most antennomeres except first antennomere; *so* only present on apical antennomere; *co* on antennomeres 5 and 6 to subapical one (absent on apical antennomere), increasing in number to maximum four on subterminal antennomere; *bo* irregular, oblate or curved, present on antennomere 6 to penultimate, increasing in number to maximum 17 on penultimate antennomere. Apical antennomere sub-spherical, somewhat wider than long (length 25–26 μm, width 33–32 μm), five *so* and 15–16 setae on distal half (Fig. 6D). All antennomeres covered with short pubescence. Chaetotaxy and sensory organs of antennae of holotype are given in Table 8.

**Table 8.** Numbers of setae and sensory organs on antennae of *Symphylella flabella* sp. nov. (holotype, excluding apical antennomere).

**Antennomere**	**Primary whorl setae**	**Secondary whorl setae**	**Rudimentary spined sensory organs**	**Cavity-shaped organs on dorsal side**	**Bladder-shaped organs**
1	6				
2	8		1		
3	9		1		
4	10		1		
5	11		1	1	
6	11			1	1
7	11	3		2	1
8	12	5		1	1
9	12	6	1	1	1
10	12	6	1		3
11	11	6	1	1	4
12	11	7	1	1	4
13	11	6	1	1	6
14	11	7	1	1	7
15	12	6	1	2	13
16	12	6	1	3	13

***Trunk*.** Length from base to tip of triangular processes somewhat shorter than or the same as its basal width; basal distance between processes of tergites distinctly longer than their length from base to tip except for tergites 2 and 3, in which basal distance shorter than length of processes (Table 9). All processes with obvious rounded swollen ends. Anterolateral setae of tergites 2, 3, 4, 6, 7, 9, 10, and 12 distinctly longer than other *lms* of same tergite, that of tergites 5, 8, 11, and 15 shorter than longest one of other *lms* of same tergite. Anterolateral setae of tergites close to length of process of same tergite. Processes with 1–3 *is.* All tergites pubescent (Fig. 5B–D).

**Table 9.** Chaetotaxy of tergites of *Symphylella flabella* sp. nov. (holotype in brackets).

Tergite	Lateromarginal setae	Inserted seta	Central setae	Other setae
1	3/4+4 (4+5)			
2	7–8 (7–8)	1–2 (2)	2 (2)	8–10 (10)
3	8–10 (9)	1–2 (1–2)	3 (3)	21–26 (26)
4	6–7 (7)	1 (1)	5 (5)	11–13 (11)
5	6–8 (7–8)	1–2 (1)	4–5 (5)	12–13 (13)
6	8–10 (10)	1–3 (2–3)	4 (4)	25–31 (31)
7	6–7 (6)	1–2 (1)	5–6 (6)	13–15 (15)
8	6–7 (6–7)	1–2 (1–2)	4–5 (4)	12–16 (16)
9	9–10 (9–10)	1–2 (2)	4–5 (5)	23–32 (28)
10	6–7 (6)	1 (1)	5–6 (6)	13–15 (15)
11	5–7 (5–6)	1–2 (1–2)	5 (5)	12–17 (17)
12	7–9 (7–8)	1–2 (1–2)	4–5 (4)	22–29 (29)
13	5–6 (6)	0–1 (0–1)	4–6 (5)	9–12 (10)
14				17–23 (21)
15	5–7 (6–7)	0–1 (0–1)	2–3 (3)	13–14 (14)
16				12–15 (12)
17				18–23 (21)

***Tergites*.** Tergite 1 reduced, with 4+5 subequal setae in holotype, 4+4 or 3+4 in paratypes (Fig. 5B). Tergite 2 complete, with two triangular posterior processes, 7–8 *lms*, 1–2 *is*, two *cs*, *als* 0.8–1.2 of length of process, processes 0.8–0.9 as long as broad, basal distance between processes 0.6–1.0 as long as their length (Fig. 5B, D). Tergite 3 complete, broader and longer than preceding one with ratios of 0.9–1.1, 0.9–1.0, and 0.8–1.2 respectively, 8–10 *lms*, 1–2 *is*, three *cs* (Fig. 5C). Tergite 4 broader than tergite 3, with ratios 1.1–1.3, 0.7–0.8, and 1.7–2.5 respectively, six or seven *lms*, one *is*, five *cs.* Chaetotaxy of tergites 5–7, 8–10, and 11–13 similar to tergites 2–4. Pattern of alternating tergite lengths of two short tergites followed by one long tergite only disrupted at caudal end (Table 10). Tergites 14, 16, and 17 without processes, with 17–23, 12–15, and 18–23 setae, respectively. Chaetotaxy and measurements of tergites are given in Tables 9, 10.

**Table 10.** Measurements of tergites and processes of *Symphylella flabella* sp. nov. (holotype in brackets, in μm).

Tergite	Length	Width	Length of processes	Basal width of processes	Basal distance between processes
1	25–30 (27)	125–145 (135)			
2	50–63 (50)	145–153 (153)	33–43 (43)	35–50 (50)	28–32 (28)
3	100–103 (100)	183–195 (195)	40–43 (43)	42–48 (48)	35–45 (35)
4	62–63 (63)	200–210 (210)	33–36 (35)	42.5–53 (53)	60–82 (70)
5	63–80 (63)	200–210 (210)	38–48 (38)	40–53 (53)	60–70 (70)
6	125–130 (125)	245–265 (265)	46–50 (48)	48–53 (53)	60–80 (60)
7	78–90 (85)	250–265 (260)	38–40 (38)	48–53 (52)	95–120 (95)
8	70–95 (70)	235–245 (245)	41–48 (48)	46–50 (50)	82–98 (82)
9	120–130 (125)	270–288 (288)	45–50 (50)	45–50 (50)	83–95 (83)
10	58–70 (70)	265–280 (280)	35–38 (38)	45–52 (52)	110–120 (110)
11	68–70 (70)	250–270 (250)	30–45 (45)	52–53 (53)	98–100 (98)
12	68–120 (68)	270–295 (270)	28–45 (28)	50–60 (60)	95–100 (100)
13	67–68 (68)	270–270 (270)	28–30 (28)	55–60 (60)	100–113 (100)
14	62–68 (68)	228–237 (228)			
15	90–100 (100)	248–255 (248)	28–30 (28)	50–57 (57)	63–75 (63)
16	50–63 (63)	200–200 (200)			
17	120–125 (125)	163–200 (163)			

***Legs*.** First pair of legs reduced to two small hairy cupules, each with one long seta (11 μm) (Fig. 5E). Basal areas of legs 2–12 each with 3–10 setae (Fig. 5F). Leg 12 almost as long as head length, trochanter 1.3–1.4× longer than wide (60–70 μm, 46–50 μm), with seven or eight subequal setae in total (Fig. 6H); femur almost as long as wide (38–45 μm, 35–40 μm), with five setae, longest dorsal seta (20–22 μm) longer than greatest diameter of podomere, pubescent dorsally, laterally with cuticular thickenings in pattern of scales; tibia nearly 1.7–1.8× longer than wide (50–55 μm, 28–30 μm), with six or seven setae, longest dorsal one (22 μm) 0.7–0.8 of greatest diameter of tibia; tarsus sub-cylindrical, 3.3–4.2× as long as wide (60–75 μm, 17–18 μm) with six dorsal setae: four straight and protruding, two depressed, longest seta (20–22 μm) slightly longer than greatest diameter of tarsus, other setae depressed and distinctly shorter. Claws slightly curved, anterior one broader and more curved than posterior one (Fig. 6I). All legs covered with dense pubescence except areas with cuticular thickenings.

***Coxal sacs*** present at bases of legs 3–9, fully developed, each with four setae on surface (Fig. 5F). Corresponding areas of legs 2, 10–12 replaced by 1–3 setae.

***Styli*** present at base of legs 3–12, with various shapes: short and wide base and fan-shaped apex in legs 3–10 (Figs 5F, 6E), while sub-conical basically and blunt, slightly swollen apex in legs 11 and 12 (Fig. 6G).

***Sense calicles*** with smooth margin around pit. Sensory seta inserted in cup center, extremely long (163–200 μm).

***Cerci*** ~ 0.7 of head in length, 3.3–3.7× as long as its greatest width (140–175 μm, 43–48 μm), densely covered with 58–71 setae (Fig. 6J, K). Two types of setae inserted on cercus: several long and erect setae located on outer and ventral sides, other setae slightly curved and depressed (Fig. 6J, K). Longest outer long and erect seta (24–25 μm) ~ 1/2 of greatest width of cerci, terminal area short (20–28 μm), circled by 8–10 layers of curved ridges (Fig. 6J, K). Terminal seta (22–28 μm) close in length with terminal area (Fig. 6J, K).

##### Etymology.

The species name *flabella* derived from the Latin word *flabellum* (fan) referring to the fan-shaped apex of styli at base of legs 3–10.

##### Distribution.

China (Chongqing).

##### Remarks.

*Symphylella flabella* sp. nov. belongs to the group of species with inserted setae present on the process of the tergite. The fan-shaped apex of styli is observed for the first time in this species of the genus.

#### ﻿ Symphylella micropora sp. nov.

Taxon classificationAnimaliaSymphylidaScolopendrellidae

2FB5A369-03E7-593C-87A7-38A43E8DEBDE

https://zoobank.org/681E58AA-5EB3-4F2C-A877-9CCA209B9B8E

**Figure 7.**
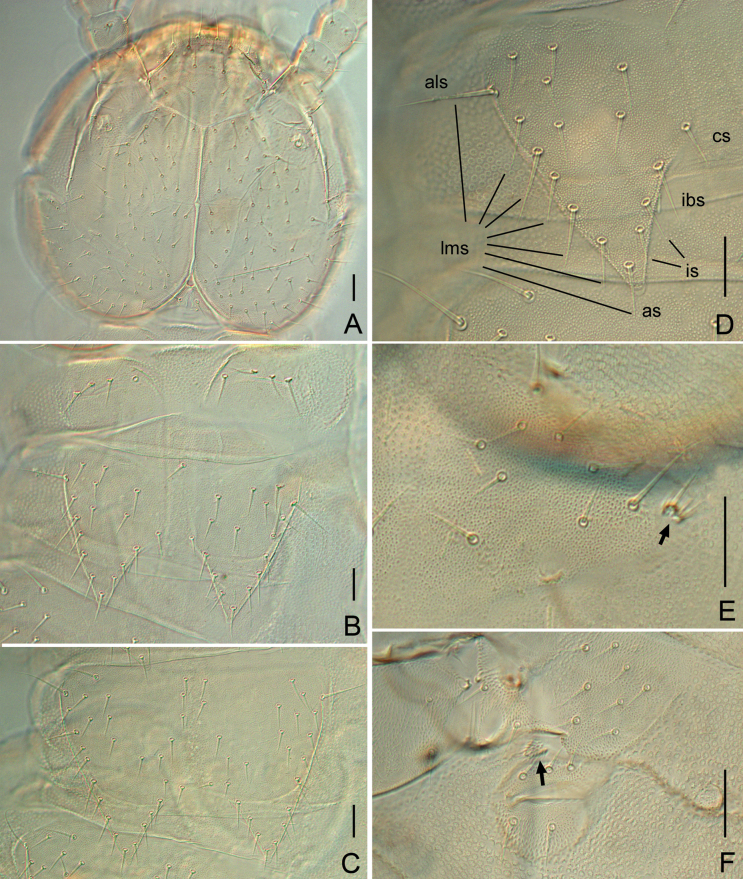
*Symphylella micropora* sp. nov. A. Head, dorsal view; B. Tergites 1 and 2; C. Tergite 3; D. Tergite 2; E. Leg 1, right side (arrow indicates reduced leg); F. Left stylus and coxal sac on base of leg 4 (arrow indicates stylus). Abbreviations: als = anterolateral seta, as = apical seta, cs = central seta, ibs = inner basal seta, is = inserted setae, lms = lateromarginal setae. Scale bars: 20 μm.

**Figure 8.**
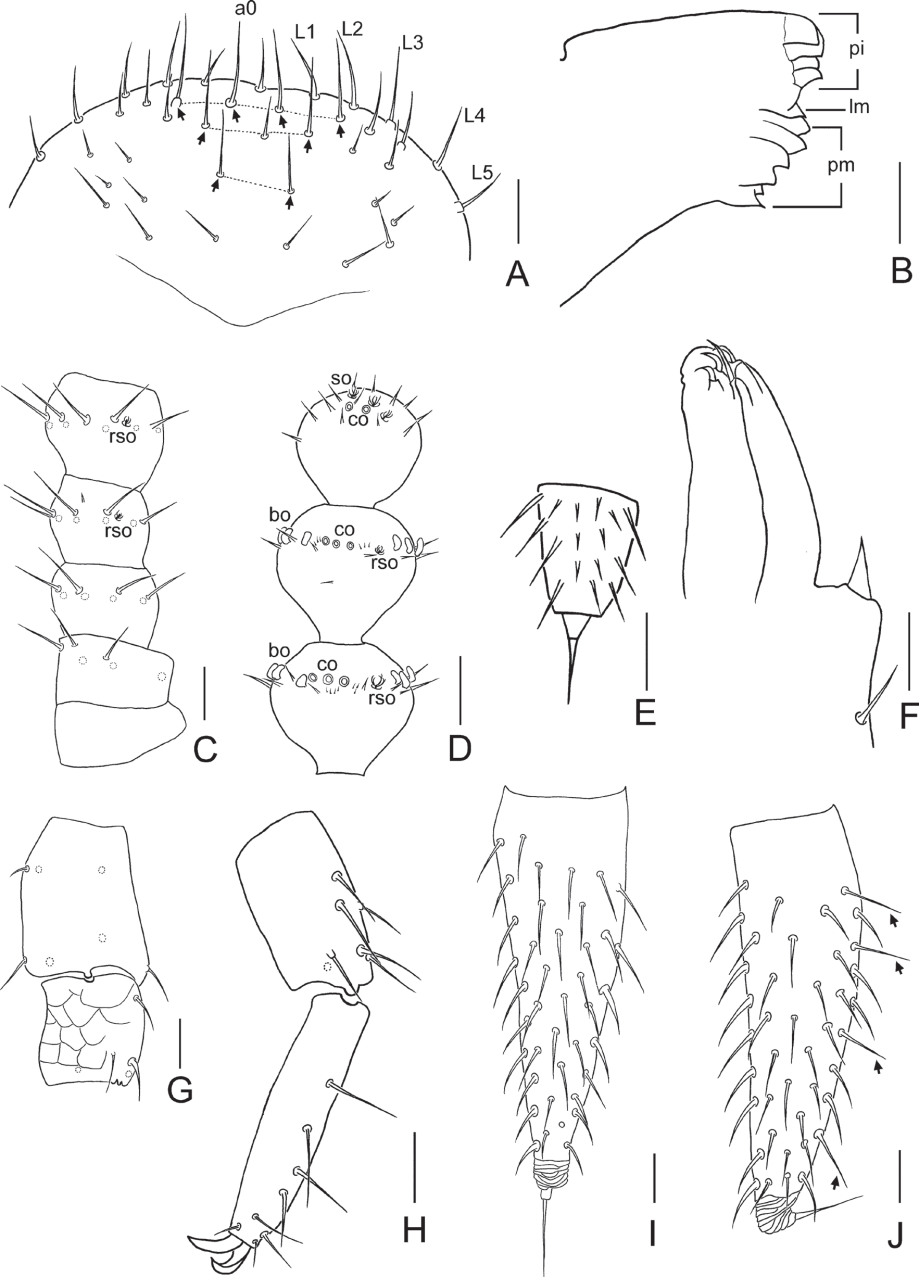
*Symphylella micropora* sp. nov. A. Frons (arrows indicate macrosetae); B. Left mandible, lateral view; C. Right 1–4 antennomeres, dorsal view; D. Right terminal three antennomeres, dorsal view; E. Right stylus at base of leg 4; F. Right first maxilla; G. Trochanter and femur of leg 12, dorsal view; H. Tibia and tarsus of leg 12, right dorsal view; I. Left cercus, dorsal view; J. Right cercus, lateral view (arrows indicate long and erect ventral setae). Abbreviations: a0 = antero-central seta, bo = bladder-shaped organ, co = cavity-shaped organ, L1–L5 = lateral setae, lm = lacinia mobilis, pi = pars incisivus, pm = pars molaris, rso = rudimentary spined sensory organ, so = spined sensory organ. Scale bars: 20 μm (A–D, F–J); 5 μm (E).

Figs 7, 8, Tables 11, 12, 13

##### Type material.

***Holotype***: • female (slide no. CQ-YTL-SY2022010) (SNHM), China, Chongqing Municipality, Wuxi County, ShuangyangTown, Yintiaoling National Natural Reserve, Daqiaowan, alt. 1022 m, 31°29'N, 109°49'E, 11-VIII-2022, coll. Y. Bu & Y. L. Jin. ***Paratypes***: • 1 female (slide no. CQ-YTL-SY2022024) (SNHM), China, Chongqing Municipality, Wuxi County, Yintiaoling National Natural Reserve, Linkouzi, alt. 1250 m, 31°28'N, 109°52'E, 14-VIII-2022, coll. Y. Bu & Y. L. Jin; • 2 females (slides no. CQ-JYS-SY2021015, CQ-JYS-SY2021029) (SNHM), China, Chongqing, Jinyunshan National Nature Reserve, extracted from soil samples of broad-leaf forest, alt. 650 m, 29°45'N, 106°21'E, 18-X-2021, coll. Y. L. Jin, Y. Bu & S.Q. Yang.

##### Other materials.

• 1 juvenile (slide no. CQ-JYS-SY2021017)(SNHM), same data as previous. • 2 juveniles (slides no. CQ-YTL-SY2022005, CQ-YTL-SY2022006)(SNHM), China, Chongqing Municipality, Wuxi County, Shuangyang Town, Yintiaoling National Natural Reserve, Hongqi, alt. 1263 m, 31°31'N, 109°49'E, 10-VIII-2022, coll. Y. Bu & Y. L. Jin. • 1 juvenile (slide no. CQ-YTL-SY2022036)(SNHM), China, Chongqing Municipality, Wuxi County, Yintiaoling National Natural Reserve, Lanying Grand Canyon, 31°26'N, 109°48'E, alt. 662 m, 16-VIII-2022, coll. Y. Bu & Y. L. Jin. • 1 juvenile (slide no. CQ-YTL-SY2022043)(SNHM), ibidem, 19-VIII-2022, coll. Y. Bu & Y. L. Jin.

##### Diagnosis.

*Symphylella micropora* sp. nov. is characterized by the distinctly small aperture of the Tömösváry organ, 4+4 setae on tergite 1, 1–3 inserted setae on processes, without swollen ends of processes, stylus with pointed apex, and long erect setae only present on the ventral side of cerci. It is most similar to *S. vulgaris* (Hansen, 1903) in the chaetotaxy of tergites, the shape of leg 12, and the chaetotaxy of the cercus. However, they differ in the chaetotaxy of tergite 1 (4+4 setae in *S. micropora* sp. nov. vs uniformly 3+3 setae in *S. vulgaris*) and the position of apical setae (not very close to the apical end in *S. micropora* sp. nov. vs very close to the apical end in *S. vulgaris*).

##### Description.

Adult body 2.2 mm long on average (2.0–2.6 mm, *n* = 4), holotype 2.1 mm.

***Head*** as long as wide, length 250–260 μm, width 240–260 μm, with widest part on equal level of points of articulation of mandibles. Central rod well developed, divided into two portions by node-like sub-middle interruption, both anterior and posterior part 60–65 μm. Dorsal side of head moderately covered with setae of different length (Fig. 7A). Frons with 5+5 lateral setae, eight macrosetae (25–30 μm) arranged as 4/2/2 and 1.5–1.9× as long as antero-central seta (a0), and 18 or 19 other setae (Fig. 8A). Cuticle on anterolateral part of head with coarse granules (Fig. 7A).

***Tömösváry organ*** globular, diameter 15–20 μm, almost half of greatest diameter of third antennomere (35–40 μm), aperture round and distinct small, 8–13 μm (Fig. 7A).

***Mouthparts*.** Mandible composed of three parts: pars incisivus (*pi*) with four distinct thick teeth, pars molaris (*pm*) with four teeth and two proximal spines, and lacinia mobilis (*lm*) with one sharp process observed from lateral view. (Fig. 8B). First maxilla typically, with sharp palp (Fig. 8F). Anterior part of second maxilla with many small protuberances, each carrying one seta, distal setae thick; posterior part with sparse setae. Cuticle of second maxilla covered with dense pubescence.

***Antennae*** with 16 or 17 antennomeres (16 in holotype), ~ 0.2 of body length. First antennomere cylindrical, 1.1–1.4× as wide as long (width 35–35 μm, length 28–33 μm), with six setae (Fig. 8C). Second antennomere wider (35–38 μm) than long (30–35 μm), with eight setae inserted around antennal wall and interior setae slightly longer than exterior ones (Fig. 8C). Chaetotaxy of third antennomere similar to second one. Setae on proximal antennomeres longer and on distal antennomeres shorter (Fig. 8C). Proximal antennomeres with only primary whorl of setae, in middle and subapical antennomeres with several minute setae in secondary whorl (Fig. 8C, D). Four types of sensory organs observed on antenna (Fig. 8D): *rso* on dorsal side from first or second to subterminal antennomeres; *so* only present on apical antennomere; *co* on antennomeres 5–6 to subapical one, increasing in number to a maximum of three on subterminal antennomere; *bo* irregular, oblate or curved, present on antennomeres 6 and 7 to penultimate, increasing in number to a maximum of 13 on penultimate antennomere. Apical antennomere sub-spherical, somewhat longer than wide (32–33 μm, 29–30 μm), five *so* and 16–18 setae on distal half (Fig. 8D). All antennomeres covered with short pubescence. Chaetotaxy and sensory organs of antennae of holotype are given in Table 11.

**Table 11.** Numbers of setae and sensory organs on antennae of *Symphylella micropora* sp. nov. (holotype, excluding apical antennomere).

**Antennomere**	**Primary whorl setae**	**Secondary whorl setae**	**Rudimentary spined sensory organs**	**Cavity-shaped organs on dorsal side**	**Bladder-shaped organs**
1	6				
2	8		1		
3	11		1		
4	11		1		
5	11	1	1	1	
6	12	5	1	2	1
7	13	4	1	1	3
8	13	7	1	2	4
9	15	6	1	1	3
10	14	8	1	2	5
11	13	7	1	2	6
12	12	8	1	2	6
13	12	9	1	2	6
14	13	7	1	2	8
15	11	7	1	1	10

***Trunk*.** Length from base to tip of triangular processes somewhat shorter than or the same as its basal width; basal distance between processes of tergites distinctly longer than their lengths from base to tip except for tergites 2 and 3, in which basal distance shorter than or the same as length of processes (Table 13). All processes without swollen ends (Fig. 7B–D). Anterolateral setae of tergites 2–4, 6, 7, 9, and 10 distinctly longer than other *lms* of same tergite, those of tergites 5, 8, 11–13, and 15 subequal or slightly shorter than longest ones of other *lms.* Anterolateral setae of tergites shorter than or subequal to process of same tergite. Processes with 1–3 *is.* All tergites pubescent (Fig. 7B–D).

***Tergites*.** Tergite 1 reduced, with 4+4 subequal setae (Fig. 7B), asymmetrically lacking one seta in two paratypes. Tergite 2 complete, with two triangular posterior processes, 6–7 *lms*, 1–2 *is*, two *cs*, *als* 0.8–0.9 of length of process, processes 0.9 as long as broad, basal distance between processes 0.8–0.9 as long as their lengths (Fig. 7B, D). Tergite 3 complete, broader, and longer than preceding one with ratios of 0.7–0.9, 0.8–0.9, and 0.8–1.5 respectively, 9–10 *lms*, 1–3 *is*, 2–3 *cs* (Fig. 7C). Tergite 4 broader than tergite 3, with ratios 1, 0.6–0.7, and 1.7–2.5 respectively, 6–8 *lms*, 1–2 *is*, 4–6 *cs.* Chaetotaxy of tergites 5–7, 8–10 and 11–13 similar to tergites 2–4. Pattern of alternating tergite lengths of two short tergites followed by one long tergite only disrupted at caudal end. Tergites 14, 16, and 17 without processes, with 19–27, 11–14, and 26–33 setae, respectively. Chaetotaxy and measurements of tergites are given in Tables 12, 13.

**Table 12.** Chaetotaxy of tergites of *Symphylella micropora* sp. nov. (holotype in brackets).

Tergite	Lateromarginal setae	Inserted seta	Central setae	Other setae
1	3+4 or 4+4 (4+4)			
2	6–7 (7)	1–2 (2)	2 (2)	10–14 (14)
3	9–10 (10)	1–3 (2–3)	2–3 (3)	21–27 (24)
4	6–8 (7)	1–2 (2)	4–6 (6)	14–19 (19)
5	7–9 (8–9)	2–3 (2–3)	4–5 (4)	13–18 (16)
6	9–12 (10–11)	2–3 (2–3)	4–5 (4)	32–40 (40)
7	6–8 (7–8)	1–2 (2)	6–7 (7)	17–24 (24)
8	7–10 (8–9)	1–3 (2–3)	4–5 (5)	11–16 (11)
9	10–12 (12)	2–3 (2–3)	4–6 (6)	30–41 (41)
10	6–7 (6–7)	1–2 (1–2)	5–7 (7)	14–20 (20)
11	7–8 (8)	1–3 (2–3)	4–6 (4)	11–17 (17)
12	7–10 (10)	1–2 (2)	4 (4)	31–32 (32)
13	4–7 (5–7)	1–2 (1–2)	4–6 (6)	8–11 (11)
14				19–27 (27)
15	6–7 (6–7)	0–2 (2)	1–2 (1)	16–23 (23)
16				11–14 (14)
17				26–33 (33)

**Table 13.** Measurements of tergites and processes of *Symphylella micropora* sp. nov. (holotype in brackets, in μm).

Tergite	Length	Width	Length of processes	Basal width of processes	Basal distance between processes
1	45–55 (50)	135–149 (141)			
2	53–65 (53)	150–192 (150)	37–42 (42)	43–45 (45)	32–37 (37)
3	95–102 (95)	193–202 (195)	43–50 (45)	48–53 (48)	35–50 (50)
4	58–63 (63)	218–235 (230)	35–38 (35)	52–57 (55)	65–88 (88)
5	63–75 (70)	73–220 (218)	43–50 (45)	45–50 (50)	70–75 (75)
6	125–140 (125)	250–280 (270)	45–55 (45)	50–75 (50)	70–88 (88)
7	58–72 (72)	257–287 (287)	35–40 (38)	45–55 (50)	100–125 (125)
8	68–75 (73)	233–255 (255)	40–53 (50)	43–58 (58)	95–107 (100)
9	108–125 (125)	262–302 (297)	47–57 (50)	50–58 (58)	75–92 (92)
10	55–70 (70)	258–298 (298)	33–40 (38)	50–55 (55)	97–120 (120)
11	60–68 (68)	225–263 (263)	42–43 (43)	50–53 (50)	80–102 (102)
12	100–113 (113)	265–287 (287)	37–48 (47)	50–60 (55)	75–95 (95)
13	55–62 (55)	240–268 (268)	25–30 (30)	45–55 (55)	87–107 (107)
14	50–57 (55)	212–237 (237)			
15	75–88 (75)	215–263 (220)	25–33 (33)	50–65 (63)	58–65 (65)
16	42–62 (42)	188–200 (200)			
17	113–125 (125)	152–192 (192)			

***Legs*.** First pair of legs reduced to two small hairy cupules, each with one long seta (10–12 μm) (Fig. 7E). Basal areas of legs 2–12 each with 2–10 setae. Leg 12 almost as long as head (Fig. 7F), trochanter 1.3–1.6× longer than wide (60–73 μm, 43–50 μm), with 5–7 subequal setae; femur slightly longer than wide (40–42 μm, 38–40 μm), with five setae (Fig. 8G), longest dorsal seta (22–23 μm) longer than half of greatest diameter of podomere, pubescent dorsally, laterally with cuticular thickenings in pattern of scales (Fig. 8G); tibia nearly 1.6–1.8× longer than wide (48–55 μm, 28–30 μm), with six or seven setae, longest dorsal one (19–20 μm) longer than greatest diameter of tibia; tarsus sub-cylindrical, 3.6–4.4× as long as wide (73–78 μm, 18–20 μm) with seven or eight dorsal setae: four straight and protruding, two depressed, longest seta (15–18 μm) close to greatest diameter of tarsus, two setae close to claw and distinctly shorter than others. Claws slightly curved, anterior one broader and more curved than posterior one (Fig. 8H). All legs covered with dense pubescence except areas with cuticular thickenings.

***Coxal sacs*** present at bases of legs 3–9, fully developed, each with 4–6 setae on surface (Fig. 7F). Corresponding areas of legs 2, 10–12 replaced by 1–5 setae.

***Styli*** present at base of legs 3–12, short and sub-conical, 8 μm in length, 5 μm in width, basal part with dense straight hairs, distal quarter hairless, with pointed apex (2–3 μm) (Figs 7F, 8E).

***Sense calicles*** with smooth margin around pit. Sensory seta inserted in cup center, extremely long (175–200 μm).

***Cerci*** length ~ 0.7 of head, 3.2–3.5× as long as its greatest width (163–175 μm, 50–53 μm), densely covered with 82–104 setae (Fig. 8I, J). Two types of setae inserted on cercus: three or four long and erect setae located only on ventral side, other setae slightly curved and depressed (Fig. 8I, J). Longest ventral seta (23 μm) not more than half of greatest width of cerci, terminal area short (20–30 μm), circled by several layers of curved ridges (Fig. 8I, J). Terminal setae (25–30 μm) longer than terminal area (Fig. 8I, J).

##### Etymology.

The species epithet *micropora* refers to the small aperture of Tömösváry organ in the new species.

##### Distribution.

China (Chongqing).

### ﻿DNA barcoding analysis

**Figure 9.**
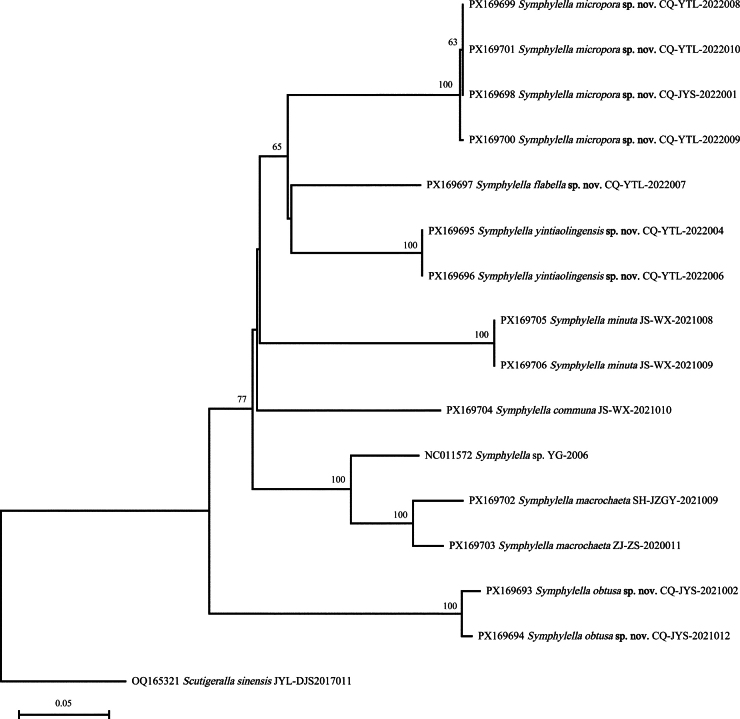
Neighbour-joining tree (JC-distance, Bootstrap 1000 replicates) of *Symphylella* inferred from COI gene sequences. Numbers on the nodes show the bootstrap values (> 50%).

All DNA barcodes newly sequenced of 14 individuals from seven *Symphylella* species are 658 base pairs in length. Each new species has a unique DNA barcode and is well separated from other congeners. The genetic distances calculated by K2P model are shown in Table 14. The results indicated that the genetic divergence between individuals of the same species is 0~4.38%, and it is 22.99% on average with a span of 8.22–30.78% between different *Symphylella* species. The higher genetic distances observed were between the species of *Symphylella* and *Scutigeralla sinensis* of the family Scutigerellidae, 32.62% on average with a span of 27.76–36.49%. The Neighbour-joining tree was constructed based on the barcoding sequences which further supported our morphological identification (Fig. 9). *Symphylella micropora* sp. nov. is recovered within a clade with *S. flabella* sp. nov. and *S. yintiaolingensis* sp. nov., whereas *S. obtusa* sp. nov. is the first diverging species among the *Symphylella* species analyzed.

**Table 14.** The pairwise distances between the species of *Symphylella* analyzed by mitochondrial COI gene (K2P model).

	Species	1	2	3	4	5	6	7	8	9	10	11	12	13	14	15	16
1	*Symphylella obtusa* sp. nov. CQ-JYS-2021002																
2	*Symphylella obtusa* sp. nov. CQ-JYS-2021012	**0.0154**															
3	*Symphylella yintiaolingensis* sp. nov. CQ-YTL-2022004	0.2991	0.2919														
4	*Symphylella yintiaolingensis* sp. nov. CQ-YTL-2022006	0.2991	0.2919	**0.0000**													
5	*Symphylella flabella* sp. nov. CQ-YTL-2022007	0.2735	0.2684	0.1453	0.1453												
6	*Symphylella micropora* sp. nov. CQ-JYS-2022001	0.3078	0.2955	0.1750	0.1750	0.1689											
7	*Symphylella micropora* sp. nov. CQ-YTL-2022008	0.3078	0.2955	0.1750	0.1750	0.1689	**0.0000**										
8	*Symphylella micropora* sp. nov. CQ-YTL-2022009	0.3078	0.2955	0.1750	0.1750	0.1709	**0.0030**	**0.0030**									
9	*Symphylella micropora* sp. nov. CQ-YTL-2022010	0.3078	0.2955	0.1750	0.1750	0.1689	**0.0000**	**0.0000**	**0.0030**								
10	*Symphylella macrochaeta* SH-JZGY-2021009	0.2961	0.2983	0.2000	0.2000	0.2190	0.2309	0.2309	0.2309	0.2309							
11	*Symphylella macrochaeta* ZJ-ZS-2020011	0.2920	0.2941	0.1942	0.1942	0.2088	0.2230	0.2230	0.2230	0.2230	**0.0438**						
12	*Symphylella communa* JS-WX-2021010	0.2799	0.2844	0.1909	0.1909	0.1890	0.2195	0.2195	0.2195	0.2195	0.2149	0.2054					
13	*Symphylella minuta* JS-WX-2021008	0.2862	0.2866	0.2129	0.2129	0.2457	0.2432	0.2432	0.2432	0.2432	0.2573	0.2389	0.2381				
14	*Symphylella minuta* JS-WX-2021009	0.2862	0.2866	0.2129	0.2129	0.2457	0.2432	0.2432	0.2432	0.2432	0.2573	0.2389	0.2381	**0.0000**			
15	*Symphylella* sp. YG-2006	0.2793	0.2772	0.1943	0.1943	0.1766	0.2002	0.2002	0.2002	0.2002	0.1062	0.0822	0.2064	0.2369	0.2369		
16	*Scutigerella sinensis* JYL-DJS2017011	0.3401	0.3322	0.3113	0.3113	0.2776	0.3416	0.3416	0.3416	0.3416	0.3195	0.2987	0.3107	0.3649	0.3649	0.2947	

Note: bold numbers are distances between individuals of the same species.

## ﻿Discussion

The genus *Symphylella* is a cosmopolitan group of symphylans (Szucsich and Scheller 2011; Bu and Jin 2018). The shapes of processes on tergites, Tömösváry organ, cerci, styli, and the chaetotaxy of head, tergites, and cerci are used as diagnostic characters for the species.

After careful observation and comparison, we found that the presence or absence of inserted setae is an ideal diagnostic character for the species when usually associated with other characters, and it was usually used in the keys to separate the species (Scheller 1971; Jin et al. 2019). The species with inserted setae generally have large body sizes (1.6–5.3 mm) and dense setae on body, while the species without inserted setae typically show small body sizes (1.2–1.8 mm) and sparse setae on body. Based on the above considerations, we propose to divide the species of the genus *Symphylella* into two groups.

**Group 1** – *isabellae*-group, characterized by the presence of inserted setae on processes of tergites, and the dense setae on head, tergites, legs and cerci. The included species are:

1. *S. adisi* Scheller, 1992
2. *S. andina* Juberthie-Jupeau, 1962
3. *S. antennata* (Hansen, 1903)
4. *S. asiatica* Scheller, 1971
5. *S. brevipes* (Hansen, 1903)
6. *S. brincki* Scheller, 1971
7. *S. capitata* Michelbacher, 1939
8. *S. caribica* Scheller, 1989
9. *S. communa* Jin & Bu, 2020
10. *S. cubae* Hilton, 1931
11. *S. delawarensis* Allen & Walther, 1993
12. *S. dunelmensis* (Bagnall, 1911)
13. *S. erecta* Domínguez Camacho & Vandenspiegel, 2012
14. *S. essigi* Michelbacher, 1939
15. *S. flabella* Jin & Bu, sp. nov.
16. *S. fuko* Domínguez Camacho & Vandenspiegel, 2012
17. *S. isabellae* (Grassi, 1886)
18. *S. jacksoni* (Bagnall, 1914)
19. *S. javanensis* Scheller, 1988
20. *S. kalundu* Domínguez Camacho & Vandenspiegel, 2012
21. *S. longiseta* Michelbacher, 1941
22. *S. longispina* Jin & Bu, 2023
23. *S. lubumbashi* Domínguez Camacho & Vandenspiegel, 2012
24. *S. macrochaeta* Jin & Bu, 2023
25. *S. macropora* Jin & Bu, 2019
26. *S. major* Scheller, 1961
27. *S. malagassa* Domínguez Camacho & Vandenspiegel, 2012
28. *S. marianensis* Scheller, 1994
29. *S. micropora* Jin & Bu, sp. nov.
30. *S. multisetosa* Scheller, 1971
31. *S. neotropica* (Hansen, 1903)
32. *S. oviceps* Michelbacher, 1939
33. *S. reddelli* Scheller, 1986
34. *S. rossi* Michelbacher, 1942
35. *S. santa* Hilton, 1931
36. *S. saratoga* Hilton, 1938
37. *S. sierrae* Michelbacher, 1939
38. *S. simplex* (Hansen, 1903)
39. *S. subantarctica* Scheller, 1974
40. *S. subterranea* Michelbacher, 1939
41. *S. tanganyika* Domínguez Camacho & Vandenspiegel, 2012
42. *S. tenella* Scheller, 1961
43. *S. tentabundna* Scheller, 1971
44. *S. texana* (Hansen, 1903)
45. *S. vulgaris* (Hansen, 1903)
46. *S. yintiaolingensis* Jin & Bu, sp. nov.
47. *S. zhongi* Jin & Bu, 2019

**Group 2** – *oligosetosa*-group, characterized by the absence of inserted setae on processes of tergites, and the sparse setae on head, tergites, legs and cerci. The included species are:^1^

1. *S. abbreviata* Scheller, 1971
2. *S. australiensis* Scheller, 1961
3. *S. bornemisszai* Scheller, 1961
4. *S. capicola* Micherbacher, 1942
5. *S. cylindrica* Scheller, 1961
6. *S. elongata* Scheller, 1952
7. *S. foucquei* Jupeau, 1954
8. *S. geum* Micherbacher, 1941
9. *S. hintoni* Edwards, 1959
10. *S. itza* Hilton, 1938
11. *S. maorica* Adam & Burtel, 1956
12. *S. minuta* Jin & Bu, 2020
13. *S. natala* Hilton, 1938
14. *S. obtusa* Jin & Bu, sp. nov.
15. *S. oligosetosa* Scheller, 1971
16. *S. plumosa* Scheller, 1971
17. *S. pusilla* (Hansen, 1903)
18. *S. tenuis* Scheller, 1961
19. *S. vaca* Hilton, 1938
20. *S. winkleri* Dobroruka, 1956

There are few DNA barcodes available for *Symphylella*, with only approximately 130 records in GenBank searched on 21 August 2025, and all samples belong to undetermined species. In the present study, we determined all samples to species level and the genetic divergences of *Symphylella* were calculated for the first time. DNA barcodes are a useful piece of supplementary evidence for morphological identification. We encourage researchers to provide molecular data as far as possible in the taxonomic study of symphylans in the future. In our Neighbour-Joining tree constructed by DNA sequences, two species *S. minuta* and *S. obtusa* sp. nov. of the *oligosetosa* group were included in the analysis; however, they were not recovered as closely related to each other. This might be caused by the limitation of short fragments of COI gene for phylogenetic reference, as well as the limited species sampled in the analysis. In order to verify the two morphological groups from a molecular perspective, more gene sequences and comprehensive sampling of species are necessary in future studies.

## Supplementary Material

XML Treatment for Symphylella

XML Treatment for Symphylella obtusa

XML Treatment for Symphylella yintiaolingensis

XML Treatment for Symphylella flabella

XML Treatment for Symphylella micropora

## Footnotes

Symphylella panama Hilton, 1939 was not listed above because of a very brief original description, and re-examination of the types is needed to clarify its position.

## ﻿Additional information

### Use of AI

No use of AI was reported.

### Author ORCIDs

Ya-Li Jin https://orcid.org/0009-0003-5773-2165

Yun Bu https://orcid.org/0000-0002-7177-9686

### Data availability

All of the data that support the findings of this study are available in the main text.
